# Genetic Consideration of Schizotypal Traits: A Review

**DOI:** 10.3389/fpsyg.2016.01769

**Published:** 2016-11-15

**Authors:** Emma E. Walter, Francesca Fernandez, Mollie Snelling, Emma Barkus

**Affiliations:** ^1^School of Psychology, University of WollongongWollongong, NSW, Australia; ^2^Illawarra Health and Medical Research Institute, University of WollongongWollongong, NSW, Australia

**Keywords:** polymorphisms, schizotypy, schizophrenia, psychosis risk, genetic disorders, family studies

## Abstract

Schizotypal traits are of interest and importance in their own right and also have theoretical and clinical associations with schizophrenia. These traits comprise attenuated psychotic symptoms, social withdrawal, reduced cognitive capacity, and affective dysregulation. The link between schizotypal traits and psychotic disorders has long since been debated. The status of knowledge at this point is such schizotypal traits are a risk for psychotic disorders, but in and of themselves only confer liability, with other risk factors needing to be present before a transition to psychosis occurs. Investigation of schizotypal traits also has the possibility to inform clinical and research pursuits concerning those who do not make a transition to psychotic disorders. A growing body of literature has investigated the genetic underpinnings of schizotypal traits. Here, we review association, family studies and describe genetic disorders where the expression of schizotypal traits has been investigated. We conducted a thorough review of the existing literature, with multiple search engines, references, and linked articles being searched for relevance to the current review. All articles and book chapters in English were sourced and reviewed for inclusion. Family studies demonstrate that schizotypal traits are elevated with increasing genetic proximity to schizophrenia and some chromosomal regions have been associated with schizotypy. Genes associated with schizophrenia have provided the initial start point for the investigation of candidate genes for schizotypal traits; neurobiological pathways of significance have guided selection of genes of interest. Given the chromosomal regions associated with schizophrenia, some genetic disorders have also considered the expression of schizotypal traits. Genetic disorders considered all comprise a profile of cognitive deficits and over representation of psychotic disorders compared to the general population. We conclude that genetic variations associated with schizotypal traits require further investigation, perhaps with targeted phenotypes narrowed to assist in refining the clinical end point of significance.

## Introduction

Schizotypal personality comprises stable behaviors characterized by persistent attenuated psychotic symptoms. These include unusual perceptual experiences, odd beliefs, unusual speech (in tone, intonation, or use of specific words), social anxiety, suspiciousness/paranoia, and isolated fully formed psychotic symptoms. The rate of clinically diagnosed schizotypal personality disorder (SPD) is approximately 4% in the general population (Pulay et al., 2009). Differentiation between schizotypal traits and SPD reflects the degree of self-recognized impairment to occupational and interpersonal functioning, frequency, and severity of symptom presentation. There are an increasing number of studies suggesting detectable reductions in interpersonal and occupational functioning in those displaying schizotypal traits (e.g., Waldeck and Miller, 2000; McGurk et al., 2013). When used as a screen in the general population, schizotypal traits are associated with depression, bipolar and affective and non-affective psychotic disorders longitudinally (e.g., Miller et al., 2002; Kwapil et al., 2013). Progression rates from SPD to a psychotic disorder are approximately 40% over 2 years (Nordentoft et al., 2006), double the risk reported in more recent studies concerned with transition from an at risk mental state (ARMS) (Fusar-Poli et al., 2013). Clinically, schizotypal traits are part of the prodrome or ARMS criteria (e.g., Salokangas and McGlashan, 2008) and SPD has now been moved from personality disorders into the Schizophrenia Spectrum and Other Psychotic Disorders section of Diagnostic and Statistical manual of Mental Disorders (DSM-V; American Psychiatric Association., 2013). The factors which determine whether someone moves from non-clinical schizotypal traits to the longitudinal expression of a clinical disorder are as yet unknown.

As well as the clinical presentation of SPD, researchers and clinicians are becoming increasingly interested in the expression of schizotypal traits or schizotypy in the general population. Clinical psychotic disorders are etiologically complex. Genetic heritability of schizotypy has been estimated around 30 to 50% within families (Claridge and Hewitt, 1987; Kendler and Hewitt, 1992; Kendler et al., 1993; Cardno et al., 1999; Chang et al., 2002; Linney et al., 2003; Macar et al., 2012). Similarly to schizophrenia susceptibility, schizotypy seems to be polygenic, with each gene likely conferring only a small to moderate risk, and interacting against background environmental factors (Grant et al., 2013; Brambilla et al., 2014). This level of complexity leads to the hypothesis that the same biological factors that underpin schizotypy in the general population are also relevant to more severe clinical disorders such as SPD and psychotic disorders. The so-called psychosis continuum suggests that risk factors for psychotic disorders will operate in a similar manner in subclinical schizotypy as in the clinical end points. Given that clinical psychotic disorders conceivably occur after a cascade and interaction between multiple biological, environmental, and psychological factors, investigating the effects of psychosis-risk-factors in subclinical schizotypes provides a window into etiological factors prior to disease progression.

During the last decade, a growing literature has emerged identifying genetic markers associated with schizotypy. We will outline the evidence for the proximity of schizotypal traits to genetic liability for psychotic disorders reporting notable specific genetic markers, such as single nucleotide polymorphisms (SNPs), associated with schizotypal traits. These studies provide evidence for population level variations in genetic bases underpinning the expression of schizotypal traits. The few linkage studies which have investigated the chromosomal regions conferring liability for schizotypal traits within families will be outlined. Collectively, these studies will delineate the evidence for schizotypal traits demonstrating heritability and co-occurrence with schizophrenia. Next, there are some genetic disorders which report rates of psychotic disorders with higher prevalence than the general population. These disorders encompass chromosomal regions which are of interest in schizophrenia. Consideration will be given to genetic disorders where expressions of schizotypal traits have been reported. These genetic disorders have well characterized genomic abnormalities and therefore further assist in pointing to chromosomal regions of significance for schizotypal trait expression.

We conducted a thorough search in the selection of articles for the current paper. We made use of PubMed, Scopus, Web of Science, PsycINFO, and Medline for the identification of articles. Key search terms were generated on the basis of the main concepts within each section, and the key words have been included in parenthesis after subsequent subheadings. All articles available in English were reviewed for inclusion. We excluded studies which considered psychotic experiences in subclinical populations, or who considered psychotic experiences after an event (e.g., stress, cannabis use). We also excluded studies which had considered the expression of schizotypal traits in the presence of bipolar risk. There is substantial debate considering the relationship between psychotic disorders and bipolar disorder which is worthy of a review in itself in order to do it justice. These publications (Mahon et al., 2013) were based within the debate of this relationship and were therefore not included in the current articles. For each section, references and similar articles were then searched to determine whether references had been missed. This was particularly relevant to the section on Schizotypal Traits and Genetic Disorders because this represented a small body of literature. Our last search of the literature was conducted in August 2016.

## Heritability and linkage studies for schizotypal traits

### Expression of schizotypal traits in families (schizotypy, schizotypal, psychosis, psychotic, family, familial, relatives, siblings, twins, parental)

The expression of schizotypal traits in relatives of patients with schizophrenia presents the opportunity to investigate whether schizotypal traits are an intermediate phenotype (Lenzenweger, 2013) for psychotic disorders. Intermediate phenotypes are heritable and underpinned by biological mechanisms. They lie between the biological risk and disease end point of interest; in this case schizotypal traits are predominantly considered for their relevance for psychosis risk. An intermediate phenotype is thought to lie mid-distance between genetic risk and the clinical end point of interest. Unsurprisingly, given we make this assumption about schizotypal traits, predisposition to schizophrenia spectrum disorders is considered to be highly heritable, demonstrated through patterns of risk in twins, and other relatives (Tsuang, 2000; Nguyen et al., 2003). First degree relatives presumably have some of the risk genes since they are at a 10-fold increased risk for developing the disorder and share approximately fifty per cent of their genes with their proband relatives. Our goal was to identify whether consistent findings pointed to a reliable relationship between familial liability and schizotypal expression. The results of schizotypal traits expressed in non-psychotic relatives of schizophrenia spectrum patients are summarized in Table 1. Preliminary findings from family studies suggest a relationship between familial-genetic liability for schizophrenia and schizotypal traits. Schizotypy has been implicated as a key expression of risk in the relationship between familial liability and schizophrenia (Lenzenweger, 2006).

**Table 1:** **Summary of expression and presence of schizotypy symptoms of studies involving relatives of Schizophrenia Spectrum probands**.

**Author**	**Schizotypy measure**	**Schizophrenia spectrum group**			**Relatives group**			**Control group**			**Group differences**
		**Disorder (Method)**	***N***	**Symptom expression mean (SD)**	**Relative type**	***N***	**Symptom expression mean (SD)**	**Control type**	***N***	**Symptom expression mean (SD)**	
Appels et al., 2004	SPQ Pos, dim. Neg. dim. Dis.	SSD (CASH)			Parents	72	Median (IQR) 2 (2.88) 5 (6.5) 1.5 (3.88)	Married couples	26	Median (IQR) 3.5 (4.13) 4.75 (9.38) 1.75 (4.00)	REL < HC ns ns
Clementz et al., 1991	PERAB PHYSAN	Scz (DSM-III)	54	6.3 (6.2) 14.3 (7.2)	Mixed	146	2.5 (3.2) 11.5 (5.6)	Non-psychiatric Comparison	178	3.8 (4.2) 8.3 (5.3)	SCZ > REL < HC SCZ > REL > HC
Grove et al., 1991	PERAB PHYSAN MMPI –sch SSP – total	Scz (DSM-III-R)	17	7.44 (7.51) 14.38 (5.74) 2.88 (1.09) 75.87 (13.84)	Mixed	61	2.25 (3.29) 13.00 (4.73) 2.41 (0.89) 47.32 (6.59)	Non-psychiatric comparison recruited through family clinic	18	1.89 (1.81) 9.61 (3.60) 2.44 (0.86) 43.94 (3.86)	SCZ > REL > HC SCZ > REL > HC ns SCZ > REL > HC
Jones et al., 2000	KSQ	Scz (SCAN for DSM-IV)	135	25.84 (13.13)	Mixed	153	9.86 (6.62)	Recruited through the Welsh Blood Transfusion Service	267	11.21 (8.4)	SCZ > REL = HC
Lataster et al., 2014	CAPE Pos, symp Neg. symp Dep. symp SIS-R Pos. schz. Neg. schz.	Scz (CASH, SCAN for DSM-IV-TR)	641	0.70 (0.51) 1.06 (0.55) 1.04 (0.59)	Siblings	811	0.020 (0.02) 0.55 (0.38) 0.63 (0.40) 0.39 (0.41) 0.28 (0.26)	Healthy controls recruited via mailing	116	0.19 (0.18) 0.48 (0.33) 0.55 (0.34) 0.46 (0.44) 0.34 (0.26)	SCZ > REL = HC SCZ > REL = HC SCZ > REL = HC REL = HC REL > HC
Mahon et al., 2013	SPQ CP ID Dis.	BPD (SCID-DSM- IV)	55	8.83 (7.6) 10.67 (7.7) 5.18 (3.17)	Siblings	53	3.65 (3.4) 5.20 (4.9) 2.33 (2.5)	Healthy controls	113	2.81 (4.1) 3.26 (4.3) 1.30 (2.2)	BPD > REL = HC BPD > REL > HC BPD > REL > HC
Solanki et al., 2012	SPQ Total CP ID Dis.	Scz (ICD-10)	‘		Mixed	50	6.70 (4.395) 2.32 (1.720) 3.06 (2.064) 1.32 (1.544)	Healthy controls recruited via word of mouth through the hospital	30	3.17 (3.312) 0.83 (1.085) 1.53 (1.655) 0.80 (1.215)	REL > HC REL > HC REL > HC ns
Vollema et al., 2002	SPQ Pos, dim. Neg. dim. Dis.	Scz	51	11.9 (8.5) 15.2 (10.3) 6.0 (5.1)	Children Siblings Parents	12 42 63	6.9 (5.3) 4.7 (4.1) 11.5 (7.8) 4.9 (4) 3.5 (3.8) 7.2 (6.1) 2.9 (3.1) 3.0 (3.5) 6.7 (5.9)				SCZ > RELS, CH > PAR, SIB > PAR SCZ > RELS SCZ > RELS, CH > PAR
Yaralian et al., 2000	SPQ Total CP ID Dis.	SSD			Mixed -family history of SSD Mixed - no family history of SSD	13 51	32.83 (16.04) 16.69 (8.35) 10.08 (7.27) 7.46 (4.56) 24.29 (13.42) 9.86 (6.68) 9.58 (6.26) 5.36 (4.29)	Psychiatric control group with family history of alcohol/drug abuse	38	27.05 (17.20) 10.79 (7.49) 10.03 (7.47) 6.28 (4.71)	ns FH+ > FH − = AD ns ns

*SPQ, Schizotypal Personality Questionnaire; SSD, Schizophrenia-spectrum disorders; CASH, Comprehensive Assessment of Symptoms and History interview; PERAB, Perceptual Aberration; PHYSAN, Physical Anhedonia; MMPI, Minnesota Multiphasic Personality Inventory; SSP, total schizotypal personality disorder rating score; KSQ, Kings Schizotypy Questionnaire; SCAN for DSM-IV, Schedule for Clinical Assessment in Neuropsychiatry for DSM-IV; SIS, Structured Interview for Schizotypy; ONAP, other non-affective psychoses; PAI, psychotic affective illness; NPAI, non-psychotic affective illness; SIS-R, Structured Interview for Schizotypy Revised; DIGS, Diagnostic Interview for Genetics Studies (psychosis section), CAPE, Community Assessment of Psychic Experiences; SAPS/SANS, Scales for Positive and Negative Symptoms; CP, Cognitive Perceptual subscale; ID, Interpersonal Deficits subscale; Dis, Disorganized subscale; BPD, Bipolar Disorder; SA, Social Anhedonia; SWAP-200, Shedler-Westen Assessment Procedure 200; TALEIA, Test for AxiaL Evaluation and Interview for clinical personnel and guidance Application*.

SPQ, Schizotypal Personality Questionnaire; SSD, Schizophrenia-spectrum disorders; CASH, Comprehensive Assessment of Symptoms and History interview; PERAB, Perceptual Aberration; PHYSAN, Physical Anhedonia; MMPI, Minnesota Multiphasic Personality Inventory; SSP, total schizotypal personality disorder rating score; KSQ, Kings Schizotypy Questionnaire; SCAN for DSM-IV, Schedule for Clinical Assessment in Neuropsychiatry for DSM-IV; SIS, Structured Interview for Schizotypy; ONAP, other non-affective psychoses; PAI, psychotic affective illness; NPAI, non-psychotic affective illness; SIS-R, Structured Interview for Schizotypy Revised; DIGS, Diagnostic Interview for Genetics Studies (psychosis section), CAPE, Community Assessment of Psychic Experiences; SAPS/SANS, Scales for Positive and Negative Symptoms; CP, Cognitive Perceptual subscale; ID, Interpersonal Deficits subscale; Dis, Disorganized subscale; BPD, Bipolar Disorder; SA, Social Anhedonia; SWAP-200, Shedler-Westen Assessment Procedure 200; TALEIA, Test for AxiaL Evaluation and Interview for clinical personnel and guidance Application

However, it is important to understand whether genetic factors alone drive elevated rates of schizotypal traits in relatives of patients with psychotic disorders. To date there are no methodologically sound population studies (with large sample sizes, controls for confounds, and for the direction of the relationship) that explore this association. There are however two studies that prospectively examine this relationship on a smaller level (Mata et al., 2000, 2003). The first published in 2000, interviewed mothers of 90 patients with schizophrenia and 121 of their psychiatrically healthy relatives with the Present State Examination (PSE; Wing et al., 1974). The authors were interested in childhood personality, social adjustment of the mothers, and schizotypal traits in relatives as assessed by the Kings Schizotypy Questionnaire (KSQ; Williams, 1993), International Personality Disorder Examination (IPDE; World Health Organization., 1992) and Venables' Survey of Attitudes and Experiences Scale (SAE; Venables et al., 1990). Positive syndrome in patients was correlated with greater scores for relatives on the three schizotypy scales; however patient syndromes did not have a relationship with their corresponding schizotypal trait (i.e., positive schizotypy to positive syndrome in patients). The second study published in 2003 sought to elucidate the link between schizotypal traits in relatives and psychopathological syndromes in patients with psychoses. Interviews were conducted with 172 patients admitted with psychosis (using the PSE) and 263 of their psychiatrically healthy relatives (using the SAE, KSQ, and IPDE). Schneiderian symptoms in patients were related to higher scores on both positive and negative schizotypal features in relatives.

These studies collectively point to schizotypal traits being highly expressed in relatives of patients with psychotic disorders. There is one point of limitation which needs to be kept in mind for studies where parent-child dyads are used in research. Given that we do not understand the heritability of schizophrenia risk, it is not possible to be certain that the parent conferring the genetic risk is included in the study (unless of course they have a diagnosed psychotic disorder themselves). This may potentially lead to weakened associations between relative dyads for schizotypal traits. Indeed, consideration needs to be given to second degree relatives and their potential role in conferring genetic liability for schizotypal traits. A study which examines schizotypal traits within an extended family, including multiple generations and first and second degree relatives, would be interesting to determine where strongest associations are found between individuals. Although these studies suggest schizotypal traits are a significant factor in the genetic risk for psychotic disorders they do not highlight specific chromosomal regions. The two studies from Mata et al. (2000) and Mata et al. (2003), suggest that profiles of symptoms in patients do not relate to their corresponding subclinical schizotypal traits, for example positive psychotic symptoms were specifically associated with positive schizotypal traits. This would suggest, schizotypal traits provide an overall risk for psychosis, rather than vulnerability toward subtypes of specific psychotic symptomatology.

### Linkage studies with schizotypal traits (schizotypy, schizotypal, psychosis, psychotic, family, familial, relatives, siblings, twins, parental, linkage, candidate, plus specific genes highlighted by review of initial literature)

Most studies which examine candidate genes involved in schizotypal genetic susceptibility are based on the results of previous studies of schizophrenia. Only a few studies have performed genetic analyses on schizotypy in relatives of patients with schizophrenia (Kendler et al., 1995; Fogelson et al., 1999; Bergman et al., 2000; Miller et al., 2002; Mata et al., 2003; Fanous et al., 2007; Lien et al., 2010). Four of these studies included the Structured Interview Schizotypy or a modified version of it to assess schizotypy in tested individuals (Kendler et al., 1995; Miller et al., 2002; Fanous et al., 2007; Lien et al., 2010). Linkage studies in families have highlighted chromosomic regions of interest related to the psychosis continuum. Existing studies suggest that both schizophrenia and schizotypal traits share some common chromosome regions (such as 4q, 5q; 6p, 6q, 8p, 9q, 10p, 10q, 11q, 15q), with an average significance generally lower in schizotypal traits than schizophrenia, regardless of ethnicity of the sample and schizotypy measure used (Levinson et al., 2000; Baron, 2001; DeLisi et al., 2002; Linney et al., 2003; Fanous et al., 2007; Xu et al., 2009; Lien et al., 2010). However, an Irish study reported higher significance schizotypal traits compared to patients with schizophrenia, highlighting chromosomal regions 5q, 9q, and 10p (Fanous et al., 2007). When an analysis was performed using a combination of both schizophrenia and schizotypy factors in a population of Taiwan relatives, both negative schizophrenia and negative schizotypal traits were linked with chromosomal region 10q22.3 while the ones for positive schizophrenia and positive schizotypy were specific to 5q14.2 and 11q23.3 (Lien et al., 2010).

From the reviewed studies, schizotypal traits do seem to be expressed at higher levels in relatives of patients with psychotic disorders compared to the general population. It is less clear whether patients and their relatives share a vulnerability to expressing the same subtypes of symptoms, such as common expression of positive trait/symptomatology. Linkage studies point to some commonality in symptom expression between patients and relatives with corresponding chromosomal regions; however the two family correlational studies did not support this. The small number of existing linkage studies, suggest the same chromosomal regions are implicated in both schizotypal traits and psychotic disorders. Subsequently the candidate genes with loci within these regions of interest are worthy of investigation in case-control studies.

## Schizotypal trait genes: association studies (schizotypy, schizotypal, psychosis, psychotic, candidate genes, dopamine, glutamate, serotonin, GABA, plus specific genes highlighted through an initial literature search)

Several schizophrenia genes have been identified through systematic fine mapping in regions implicated by linkage analysis (Liu et al., 2002) and through follow up analysis of linkage peaks to systematically identify candidate genes (Hennah et al., 2003; Duan et al., 2004; Petryshen et al., 2005; Pimm et al., 2005). Due to the small number of linkage studies investigating schizotypal traits, the candidate genes previously associated with schizophrenia drive the focus of SNP selection in schizotypal trait studies. For a disorder such as schizophrenia, which appears to be genetically complex, genetic markers including SNPs provide a plausible mechanism to underpin the expression of attenuated intermediary phenotypes such as schizotypal traits to be presented within a healthy population. An inherent assumption in this area is that each genetic marker will confer a degree of risk for the expression of psychotic disorders, and it is an accumulation of risk alleles in a dosing manner which sets background genetic liability. Overall results from literature search of schizotypy and candidate genes can be found in Table 2.

**Table 2:** **Summary of SNP studies for schizotypy**.

**Gene**	**Author**	**Marker**	**Study design**	***N*** **(% male)**	**Origin/Ethnicity**	**Schizotypy measure**	**Association**
AHI1	Leach et al., 2013	rs1154801	Cross-sectional GWAS—healthy university sample	519 (36%)	Caucasian	SPQ-BR	No significant association
		rs2064430					No significant association
AKT1	Leach et al., 2013	rs3803300	Cross-sectional GWAS—healthy university sample	519 (36%)	Caucasian	SPQ-BR	No significant association
BDNF	Ma et al., 2007	rs6265	Representative healthy community sample	465 (49%)	China	SPQ	No significant association
C6orf217	Leach et al., 2013	rs10223338	Cross-sectional GWAS—healthy university sample	519 (36%)	Caucasian	SPQ-BR	No significant association
CACNA1C	Roussos et al., 2013	rs1006737	Case-controls in two-wave GWAS—healthy controls vs SPD patients	48 HC 50 SPD	Greece	STQ SIDP- IV	Association with paranoid ideation and (*p* = 5.0 × 10^−4^) and unusual experiences (*p* = 0.03). The A allele increased the risk for SPD (*p* = 0.03, OR = 1.91) and was associated at a trend level with paranoia in the SPD patient group (*p* = 0.053)
CCKAR	Leach et al., 2013	rs1800857	Cross-sectional GWAS—healthy university sample	519 (36%)	Caucasian	SPQ-BR	No significant association
CNR1	Arias et al., 2010	rs1049353	Cross-sectional GWAS—healthy undergraduates	451 (44%)	Spain	SPQ-B, CAPE	No significant association
CNR2	Arias et al., 2010	rs16828926	Cross-sectional GWAS—healthy undergraduates	451 (44%)	Spain	SPQ-B, CAPE	No significant association
COMT	Arias et al., 2010	rs4680	Cross-sectional GWAS—healthy undergraduates	451 (44%)	Spain	SPQ-B, CAPE	No significant association
	Avramopoulos et al., 2002	rs4680	Cross-sectional GOI—healthy air force recruits	379 (100%)	Greece	SPQ, PAS	Those homozygous for high activity allele had significantly higher scores on the SPQ (*p* = 0.024) and the PAS (*p* = 0.005)
	de Castro-Catala et al., 2015	rs4680	Cross-sectional GOI	808 (23%)	Spain	WSS, CAPE	Association with higher negative dimension scores on WSS (*p* = 0.024) and CAPE (*p* = 0.004) for males carrying Val alleles only
	Grant et al., 2013	rs4680	Cross-sectional GWAS	288 (31%)	Germany	O-LIFE	Association with full (*p* = 0.092) and short (*p* = 0.031) scale scores for unusual experiences
	Ma et al., 2007	rs4680	Representative healthy community sample	465 (49%)	China	SPQ	Significant influence on total SPQ (*p* = 0.013), disorganization (*p* = 0.042) and constricted affect (*p* = 0.027) for males only
		rs165599					No significant association
	Schürhoff et al., 2007	rs4680	GOI case-control—first degree relatives of probands vs healthy controls	106 (47%)	Caucasian	SPQ	High activity allele (Val) was associated with higher cognitive perceptual (*p* = 0.001) and interpersonal deficit (*p* = 0.04) scores. Additionally, those homozygous in Val were associated with higher total SPQ (*p* = 0.01) scores
	Smyrnis et al., 2007	rs4680	Cross-sectional GOI—healthy air force recruits	1657 (100%)	Greece	SPQ, PAS	Association with total SPQ (*p* = 0.03) and negative (*p* = 0.04), disorganized (*p* = 0.02) and paranoid *p* = 0.03) factors of the PAS
	Zammit et al., 2014	rs2097603	Longitudinal general population representative GWAS—adolescents	3483	UK	PLIKSi	Association with definite psychotic experiences, however no longer present following correction for multiple tests
DAAO	Stefanis et al., 2007	rs2111902	Cross-sectional GWAS—healthy military conscripts	2076 (100%)	Greece	SPQ, PAS, CAPE	No significant association
		rs3918346					No significant association
		rs3741775					No significant association
DAOA	Leach et al., 2013	rs3916971	Cross-sectional healthy university sample	519 (36%)	Caucasian	SPQ-BR	No significant association
	Stefanis et al., 2007	rs2391191	Cross-sectional GWAS—healthy military conscripts	2076 (100%)	Greece	SPQ, PAS, CAPE	No significant association
		rs778293					No significant association
		rs3918342					No significant association
DISC1	Leach et al., 2013	rs999710	Cross-sectional healthy university sample	519 (36%)	Caucasian	SPQ-BR	No significant association
DRD1	Gurvich et al., 2016	rs778293	Cross-sectional GOI—healthy individuals	127	Mixed	O-LIFE	Significant association with negative dimensions scores (*p* = 0.010) for the minor rs4532/C allele
DRD2	Grant et al., 2013	rs1800497	Cross-sectional GWAS	288 (31%)	Germany	O-LIFE	Association of the A1 allele with impulsive nonconformity scores for males only
	Leach et al., 2013	rs6275	Cross-sectional GWAS—healthy university sample	519 (36%)	Caucasian	SPQ-BR	No significant association
		rs6277					No significant association
	Montag et al., 2015	rs1800497	Cross-sectional GWAS—healthy university sample	471	Caucasian	SPQ-B	No significant association
	Taurisano et al., 2014	rs1076560	Cross-sectional GOI—healthy adults	83	Caucasian	SPQ	Greater total schizotypy scores associated with minor T allele (*p* = 0.008)
DRD3	Montag et al., 2015	rs6280	Cross-sectional GWAS—university sample	471	Caucasian	SPQ-B	No significant association
DTNBP1	Leach et al., 2013	rs1474605	Cross-sectional GWAS—healthy university sample	519 (36%)	Caucasian	SPQ-BR	No significant association
		rs3213207					No significant association
	Stefanis et al., 2007	rs760761	Cross-sectional GWAS—Healthy military conscripts	2076 (100%)	Greece	SPQ, PAS, CAPE	Association with lower scores on the SPQ dimensions of paranoia (*p* = 0.007) and cognitive-perceptual disturbances (*p* = 0.034) for the minor allele
		rs2619522					Association with lower scores on the SPQ dimensions of paranoia (*p* = 0.005), disorganization (*p* = 0.018) and cognitive-perceptual disturbances (*p* = 0.034) for the minor allele
		rs1018381					Association with lower scores on the SPQ paranoia dimension (*p* = 0.026), and positive factor of CAPE (*p* = 0.043) for the minor allele
		rs2619539					No significant association
		rs3213207					No significant association
		rs1011313					No significant association
		rs2005976					No significant association
ERBB4	Stefanis et al., 2011	rs707284	Cross-sectional GWAS—healthy military conscripts	1127 (100%)	Greece	SPQ	No significant association
		rs839523					No significant association
		rs7598440					No significant association
	Zammit et al., 2014	rs4673628	Longitudinal general population representative GWAS—adolescents	3483	UK	PLIKSi	Association with definite psychotic experiences, however no longer present following correction for multiple tests
FAAH	Arias et al., 2010	rs324420	Cross-sectional GWAS—healthy undergraduates	451 (44%)	Spain	SPQ-B, CAPE	Interaction between cannabis use and higher scores on both SPQ-B-disorganized dimension (*p* = 0.013) and CAPE- negative dimension (*p* = 0.034) for A allele
GABRB2	Leach et al., 2013	rs1816072	Cross-sectional GWAS—healthy university sample	519 (36%)	Caucasian	SPQ-BR	No significant association
GNBIL	Zammit et al., 2014	rs2269726	Longitudinal general population representative GWAS—adolescents	3483	UK	PLIKSi	Association with definite psychotic experiences, however no longer present following correction for multiple tests
BRM3	Zammit et al., 2014	rs6465084	Longitudinal general population representative GWAS—adolescents	3483	UK	PLIKSi	Association with definite psychotic experiences, however no longer present following correction for multiple tests
GWA_11p14.1	Leach et al., 2013	rs1602565	Cross-sectional GWAS—healthy university sample	519 (36%)	Caucasian	SPQ-BR	No significant association
GWA_16p13.12	Leach et al., 2013	rs7192086	Cross-sectional GWAS—healthy university sample	519 (36%)	Caucasian	SPQ-BR	No significant association
HIST1H2BJ	Leach et al., 2013	rs6913660	Cross-sectional GWAS—healthy university sample	519 (36%)	Caucasian	SPQ-BR	Risk allele carrier greater cognitive-perceptual scores (*p* = 0.0134). Became non-significant after correcting for multiple tests
HTR2A	Leach et al., 2013	rs6311	Cross-sectional GWAS—healthy university sample	519 (36%)	Caucasian	SPQ-BR	No significant association
MAOA	Grant et al., 2013	MAOA-uVNTR	Cross-sectional GWAS	288 (31%)	Germany	O-LIFE	Low activity group associated with greater scores for inattentive anhedonia for both full (*p* = 0.016) and short (*p* = 0.021) scales, for males only
MAGI2	Zammit et al., 2014	rs6951046	Longitudinal general population representative GWAS - adolescents	3483	UK	PLIKSi	Association with definite psychotic experiences, however no longer present following correction for multiple tests
MDGA1	Leach et al., 2013	rs11759115	Cross-sectional GWAS—healthy university sample	519 (36%)	Caucasian	SPQ-BR	No significant association
		rs12191311					No significant association
MIR137	Zammit et al., 2014	rs1625579	Longitudinal general population representative GWAS - adolescents	3483	UK	PLIKSi	Association with definite psychotic experiences—risk allele reduced in frequency
NOTCH4	Leach et al., 2013	rs2071287	Cross-sectional GWAS—healthy university sample	519 (36%)	Caucasian	SPQ-BR	No significant association
NRG1	Leach et al., 2013	rs10503929	Cross-sectional GWAS—healthy university sample	519 (36%)	Caucasian	SPQ-BR	Risk allele carrier greater cognitive-perceptual scores (*p* = 0.0473). Became non-significant after correcting for multiple tests
	Stefanis et al., 2007	SNP8NRG221132	Cross-sectional GWAS - healthy military conscripts	2076 (100%)	Greece	SPQ, PAS, CAPE	No significant association
		SNP8NRG221533					No significant association
		SNP8NRG241930					No significant association
		SNP8NRG243177					No significant association
		SNP8NRG433E1006					No significant association
NRGN	Leach et al., 2013	rs12807809	Cross-sectional GWAS—healthy university sample	519 (36%)	Caucasian	SPQ-BR	No significant association
NRXN1	Zammit et al., 2014	rs3850333	Longitudinal general population representative GWAS - adolescents	3483	UK	PLIKSi	Association with definite psychotic experiences, however no longer present following correction for multiple tests
NT5C2	Zammit et al., 2014	rs11191580	Longitudinal general population representative GWAS - adolescents	3483	UK	PLIKSi	Association with definite psychotic experiences—risk allele reduced in frequency
PDE4B	Leach et al., 2013	rs910694	Cross-sectional GWAS—healthy university sample	519 (36%)	Caucasian	SPQ-BR	Risk allele carrier lower total schizotypy scores (*p* = 0.0475) Became non-significant after correcting for multiple tests
PPP3CC	Leach et al., 2013	rs10108011	Cross-sectional GWAS—healthy university sample	519 (36%)	Caucasian	SPQ-BR	No significant association
PRODH	Ma et al., 2007	rs385440	GOI - representative healthy community sample	465 (49%)	China	SPQ	No significant association
		rs372055					No significant association
PRSS16	Leach et al., 2013	rs13219354	Cross-sectional GWAS—healthy university sample	519 (36%)	Caucasian	SPQ-BR	No significant association
		rs6932590					Risk allele carrier greater cognitive-perceptual scores (*p* = 0.0363) and greater total schizotypy scores (*p* = 0.0266). Becomes non-significant after correcting for multiple tests
RELN	Leach et al., 2013	rs262355	Cross-sectional GWAS—healthy university sample	519 (36%)	Caucasian	SPQ-BR	Risk allele carrier greater interpersonal (*p* = 0.0118) and disorganized scores (*p* = 0.0401). Becomes non-significant after correcting for multiple tests
		rs7341475					No significant association
RGS4	Leach et al., 2013	rs2661319	Cross-sectional GWAS—healthy university sample	519 (36%)	Caucasian	SPQ-BR	Risk allele carrier lower interpersonal (*p* = 0.0162), cognitive-perceptual (*p* = 0.0125), and total schizotypy scores (*p* = 0.0155). Becomes non-significant after correcting for multiple tests
	Stefanis et al., 2008	rs2661319	Cross-sectional GWAS - healthy military conscripts	1127 (100%)	Greece	SPQ	Associated with greater SPQ scores (*p* = 0.039) for the A allele
		rs951436					Association with greater negative SPQ scores (*p* = 0.009) for the T allele
		rs951439					No significant association
		rs10917670					No significant association
RPP21	Leach et al., 2013	rs3130375	Cross-sectional GWAS—healthy university sample	519 (36%)	Caucasian	SPQ-BR	No significant association
SLC6A3	Grant et al., 2013	DAT 3′ UTR-VNTR	Cross-sectional GWAS	288 (31%)	Germany	O-LIFE	Cognitive distortion score associated with 9-repeat-allele in males only (small sample)
TPH1	Leach et al., 2013	rs1799913	Cross-sectional GWAS—healthy university sample	519 (36%)	Caucasian	SPQ-BR	No significant association
ZNF804A	Leach et al., 2013	rs1344706	Cross-sectional GWAS—healthy university sample	519 (36%)	Caucasian	SPQ-BR	No significant association
	Stefanis et al., 2013	rs7597593	Cross-sectional GWAS - healthy military conscripts	1507 (100%)	Greece	SPQ, PAS, CAPE	Significant association with paranoid SPQ scores (*p* = 0.004). Additionally, an association trend with positive factors for the CAPE and PAS for individuals with the major C allele (*p* = 0.081). The A risk allele was associated with decreased scores in SPQ factors as well as the PAS and CAPE positive factor
		rs1344706					Significant association with paranoid (*p* = 0.02) and disorganization (*p* = 0.081) SPQ scores. The T risk allele was associated with decreased scores in SPQ factors as well as the PAS and CAPE positive factor
		rs4667001					No significant association
		rs3731834					No significant association
	Yasuda et al., 2011	rs1344706	Cross-sectional GOI - healthy individuals	176 (47%)	Japan	SPQ	Association with higher total SPQ scores (*p* = 0.042) and disorganization scores (*p* = 0.033) for the risk T allele

*SPQ BR, Schizotypal Personality Questionnaire Brief Revised; SPQ, Schizotypal Personality Questionnaire; STQ, Schizotypal Traits Questionnaire; SIDP- IV, Structured Interview for DSM-IV Personality; CAPE, Community Assessment of Psychic Experiences; SPQ B, Schizotypal Personality Questionnaire Brief version; PAS, Perceptual Aberration Scale; WSS, Wisconsin Schizotypy Scales; O-LIFE, Oxford-Liverpool Inventory of Feelings and Experiences; PLIKSi, Psychosis-Like Symptoms interview*.

Among these candidate genes, dystrobrevin-binding protein 1 gene *(DTNBP1)* located on chromosome 6q22.3 has been associated with schizophrenia in diverse populations (Bray et al., 2005; Tochigi et al., 2006; Tosato et al., 2007; Vilella et al., 2008). This gene codes for a synaptic protein dysbindin 1 which is found presynaptically. Dysbindin seems to be involved in the exocytotic glutamate release and cognition processes, in particular memory (Burdick et al., 2006; Talbot et al., 2006; Bhardwaj et al., 2009; Hashimoto et al., 2010). SNPs within this gene are associated with lower levels of dysbindin expression in post-mortem brain studies (Talbot et al., 2004, 2006), increased risk for schizophrenia in case-control studies (Straub et al., 2002) and with paranoid schizotypy in a Caucasian sample (Stefanis et al., 2008). In reference to glutamatergic functioning, P250 protein is also important given that it plays a significant part in N-methyl-D-aspartate receptor (NMDAR) mediated spine development. This protein was found to be highly enriched in the post-synaptic densities of neurons and is co-localized with the NR2B subunit of the NMDAR. NDMAR plays an essential role in the synaptic plasticity and memory performance. SNPs in the P250 gene have been previously proposed to be involved in schizophrenia vulnerability (Ohi et al., 2012). In a Japanese cohort, genetic variant (rs2298599) in the P250 gene was also found to be significantly associated with high scoring of schizotypal traits. NRG1 function is mediated by a class of receptor tyrosine kinases including erbB4 (Li et al., 2007), and has been shown to associate with schizophrenia (Law et al., 2007) with altered NRG1/erbB4 signaling being reported in the brains of schizophrenia patients (Hahn et al., 2006). Two studies have investigated SNPs within this gene (Stefanis et al., 2011; Zammit et al., 2014), and results have been inconsistent with only one SNP (rs4673628) associating with positive schizotypy.

Genes involved in the regulation of dopamine have been a strong candidate due to dopamine's documented role in schizophrenia and schizotypal traits (e.g., Woodward et al., 2011; Seeman and Seeman, 2014). Considerable attention has been given to the catechol-O-methyltransferase gene (or COMT). This gene codes for an enzyme involved in the breakdown of catecholamines, including dopamine, at the synapse. A single variation from G to A at codon 158 of COMT (rs4680) results in a valine (Val) to methionine (Met) substitution in the amino acid sequence of the protein. This change leads to a functional alteration in the activity of the enzyme: Low (Met), Medium (Val/Met) and High (Val). Several studies have reported an association between high activity allele and patients with schizophrenia (Glatt et al., 2003; Gogos and Gerber, 2006). Authors suggest that the high activity enzyme leads to lower dopamine activity in the cortex and can account for both the negative and cognitive symptoms found in schizophrenia. This SNP has been associated with higher schizotypy scores in several gene-wide association studies (GWAS) (Caucasian population: Avramopoulos et al., 2002; Schürhoff et al., 2007; Smyrnis et al., 2007; Grant et al., 2013; de Castro-Catala et al., 2015. Chinese population: Ma et al., 2007) but not all (Caucasian population: Arias et al., 2010; Grant et al., 2014; Zammit et al., 2014). A key regulator of the levels of dopamine in the synapses in brain is the dopamine transporter protein (DAT), encoded by gene SLC6A3 (located at 5p15.3). Unlike COMT which operates within the synapse, DAT reuptakes the neurotransmitter into the presynaptic neuron. Given the functional importance to neurotransmission of this gene, it has been comparatively under investigated in relation to schizotypy. The functional polymorphism of interest is located at the 3′UTR region of SLC6A3 in a form of a Variable Number Tandem Repeat (VNTR) of 40 bp. Rare allele 9 carriers of this VNTR were found with schizotypal features (Grant et al., 2013), however due to the limited sample this result has to be considered with caution. Rather than just focusing on the factors involved in breaking down or taking up neurotransmitters, attention has also been given to SNPs coding for dopamine receptors. These SNPs can lead to functional and structural alterations in dopamine (e.g,. D'Souza and Craig, 2006; Michealraj et al., 2014). Gurvich et al. (2016) found that dopaminergic receptor D1 was linked to increased scores on schizotypy factors that resemble negative symptoms in clinically diagnosable schizophrenia, while Grant et al. (2014) reported that SNPs in the dopaminergic receptors D4 and D5 were negatively correlated with schizotypal features. These authors concluded that schizotypy is associated with dopamine dysregulation. However, the results for dopamine receptors as a whole are mixed, with Grant et al. (2013) and Taurisano et al. (2014) finding an association with schizotypy at the D2 receptor, which conflicts with the findings of Leach et al. (2013) and Montag et al. (2015).

Several other functional SNPs with relevance to neurotransmission and brain maturation have also been investigated in relation to schizotypy. A factor considered essential for supporting neuronal growth and promoting neuronal survival is brain-derived neurotrophic factor (BDNF). BDNF has been widely investigated for its relevance to schizophrenia (Kheirollahi et al., 2016). Proline is an amino acid which is found in excess in patients with schizophrenia. There is a SNP of interest in the gene coding for proline oxidase enzyme, which is able to metabolize (PRODH). Additionally, a SNP coding for D-Amino Acid Oxidase enzyme involved in glutamatergic neurotransmission (DAAO), has been demonstrated to be associated with schizophrenia in case-control studies (Caldinelli et al., 2013). One study in a Chinese population (Yang et al., 2013), found no association between these SNPs and schizotypy scores. Similarly, after correction for multiple comparisons, no significant associations were observed between SNPs in DAAO gene and schizotypy in a Greek military healthy cohort (Stefanis et al., 2007). The same cohort was employed to evaluate risk factors for schizotypy for selected SNPs in RGS4 gene. The RGS4 gene codes for Regulator of G protein signaling, which is involved in intracellular signaling in glutamatergic and dopaminergic receptors. Genetic variant for rs951436 and rs2661319 were associated with negative schizotypy and total schizotypy respectively, however none of the tested SNPs were associated with cognitive indices, despite the seeming functional importance of RGS4 (Stefanis et al., 2008).

Looking at other candidate neuronal receptors, genetic variants for CACNA1C gene has been of interest to some investigators. This gene codes for a sub-unit of the calcium channel which regulates the calcium influx into the cells; a mechanism which has implications for learning and memory. One SNP in this gene (rs1006737) has been previously associated with schizophrenia (Porcelli et al., 2015). In a Caucasian sample, schizotypal paranoid ideation was associated with this SNP (Roussos et al., 2011). In another study from the same group, the same genetic variant was positively associated with interview assessed schizotypal personality disorder paranoid ideation and unusual experiences (Roussos et al., 2013). Given the replication of an association between schizotypal traits and this SNP it is worthy of investigation in future studies.

An increasing area of exploration in association studies is the zinc finger (ZNF) family. The ZNF family plays roles in the binding of DNA- and RNA proteins; where amino acids are folded into a single structural unit around a zinc atom to stabilize the fold (Sun et al., 2015). They are involved in multiple functions, including transcriptional activation, protein folding, RNA packaging, lipid binding, and DNA recognition (Laity et al., 2001). Recently, focus has been given to specific SNPs of ZNF804A (e.g., rs1344706, rs4667001, and rs728534) with a meta-analysis by Zhu et al. (2014) showing an association with schizophrenia risk in both Asian and Caucasian populations. When investigating these SNPs in reference to schizotypy, we see mixed results with some studies finding a significant association with schizotypy (Yasuda et al., 2011; Stefanis et al., 2013) and others not (Leach et al., 2013).

There is burgeoning interest in whether genes inherited from the maternal and paternal lines have differing effects, known as imprinting. This has been partly driven by consistent findings for increasing paternal age being related to psychosis risk (Foutz and Mezuk, 2015; Martin et al., 2015) and speculation on what mechanism may be underpinning this. Additionally, the role of imprinting in psychiatric genetics is becoming more widely speculated upon and a point for consideration within psychotic disorders (Crespi, 2008). In support of this the imprinted gene LRRTM1 (Leucine-rich repeat transmembrane neuronal 1) was reported to be a risk factor for mental illness such as schizophrenia when the gene was inherited from the father (Francks et al., 2007). LRRTM1 plays a significant role in brain development and reflects a variable profile of maternal regulation (Francks et al., 2007). Given that schizophrenia and schizotypal traits are considered to be neurodevelopmental, this gene may be of functional significance. Three SNPs (rs1007371, rs1446109, rs723524) in the LRRTM1 gene were examined in a student Canadian cohort for their association with a brief measure of schizotypy (Leach et al., 2014). However, none of the tested SNPs were significantly associated with schizotypy after correction for multiple comparisons. Given the gene's significance for neurodevelopment and maturation of the brain, potentially through the lifespan, this requires further investigation.

To some extent linkage studies have led to SNPs previously associated with schizophrenia being investigated for their relevance to schizotypal traits. The findings from the SNP studies are mixed, at best. However, they are similarly mixed using patient groups, where a concentrated expression of biological risk is found. The advantage of examining schizotypy in healthy populations is the potential to achieve large sample sizes, often compromised in patient case-control studies. However, there are caveats to such an approach. First the genetic risk will be “watered-down” in the attenuated schizotypal traits. Assuming a polygenic approach to risk, this will mean each individual will carry fewer at risk alleles. Secondly, there is as much heterogeneity in the expression of schizotypal traits in the healthy population as there is in the presentation of symptoms in patient populations. Thirdly, it is possible that schizotypes also carry protective SNPs which decrease the likelihood of transition to psychosis, this complicates a scenario which we know represents the convergence of multifaceted risk markers, only some of which are biological in nature. To date, few studies have taken advantage of the possibility of using large sample sizes to investigate the genetic underpinnings of schizotypy. There may be both pragmatic and theoretical concerns at play. Some still appear to question the utility of schizotypy to investigate psychotic disorders, questioning its role in risk for psychosis as well as the possibility it represents a useful intermediate phenotype. Others question the degree to which it is of interest in itself. These continued concerns being voiced in the literature ensure that obtaining funding to investigate the biological underpinnings of schizotypy can be difficult; restricting the investigation of the genetic underpinnings to often already established population databases where measures are limited and phenotypic refinement not possible. Finally, there are sample overlaps in the studies summarized here. Multiple research groups need to investigate these questions, each with their own approach to ensure that thorough and representative research eventuates in the future.

## Genetic disorders reporting schizotypal traits (schizotypy, schizotypal, psychosis, psychotic, schizophrenia, genetic disorders, developmental disorders)

Alongside the traditional methods of linkage, family studies and SNPs examining the underpinnings of the psychosis continuum, an alternative approach exists. There are a number of genetic disorders, with well-characterized genetic deletions or insertions, where rates of schizophrenia are considered to be higher than those found in the general population. In these instances, the genetic mutations are established, often with documented physical and psychological implications. It is possible that consideration of these disorders in relation to schizotypal traits would assist in providing additional evidence for the biological mechanisms underpinning the expression of these traits. Interestingly, given the current diagnostic manuals, individuals with well-defined genetic variations cannot receive a diagnosis of schizophrenia. However, some debate surrounds whether psychotic symptom presentation in those with genetic variations differs from the strict definition of schizophrenia (e.g., Bassett et al., 2003). A brief overview of genetic disorders with elevated rates of schizophrenia, where consideration has been given to schizotypal traits will be outlined.

Perhaps the most well-known genetic disorder associated with psychotic disorders is Chromosome 22q11.2 deletion syndrome. Deletion of 22q11.2 underpins a group of syndromes including DiGeorge syndrome, velocardiofacial syndrome (VCFS; also called Shprintzen syndrome), Opitz G/BBB syndrome and Conotruncal anomaly face syndrome. Chromosome 22q11.2 deletion syndrome is associated with physical problems in the cleft palate, heart, kidneys, lungs, autoimmune problems, and distinctive facial features. Many individuals have lower IQ than the general population, are developmentally delayed and have cognitive deficits. The deletion occurs in 1:5000 live births (Botto et al., 2003); although some argue rates may be much higher, given the heterogeneity of presentation it is possible those with the deletion will receive an alternative diagnosis and may not receive genetic testing. There is substantial evidence documenting elevated rates of schizophrenia in adolescence and adulthood in those with 22q11.2 deletion syndrome when compared to the general population. In addition, they appear to be at greater risk for developing schizophrenia than other learning disabled individuals (Gothelf et al., 2007). The existence of subclinical psychotic symptoms in children and adolescents with 22q11.2 deletion syndrome has clinical predictive validity for future psychotic disorders (Gothelf et al., 2007). Indeed, Debbané et al. (2006) reported 28% of their child and adolescent sample had psychotic symptoms. Consequently, the examination of schizotypal features in 22q11.2 deletion syndrome individuals is clinically valid as a potential preventative measure in this group as well as providing additional information concerning the pathogenesis of the psychosis.

Monks et al. (2014) reported rates of schizotypy to be higher in a 22q11.2 deletion sample compared to controls, even when participants with a current psychotic disorder were excluded from the 22q11.2 deletion group. Adolescents with 22q11.2 deletion syndrome have been reported to have elevated interpersonal schizotypal traits compared to controls, and the assessment of these traits appeared to be consistent over a three year period (Fonseca-Pedrero et al., 2016). In addition, higher levels of negative schizotypal traits have been reported in those with 22q11.2 deletion when compared to controls, even after controlling for anxiety, depression and full scale intelligence (Schneider et al., 2015). There are a number of genes captured by the 22q11.2 deletion area, these include: COMT, PRODH, zinc finger gene (ZDHHC8) and armadillo repeat protein deleted in velocardiofacial syndrome (ARVCF; see Harrison and Weinberger, 2005; Prasad et al., 2008 for reviews). Polymorphisms in these genes have been varyingly associated with cognitive deficits, schizophrenia, high schizotypy and psychotic symptoms both in psychiatric populations and in 22q11.2 samples. However, these associations have not been consistently reported; and, to date, it is difficult to determine whether negative findings are due to sample differences (e.g., cultural variation in allelic frequency), sex differences, phenotypic heterogeneity or low statistical power.

A disorder which has also been investigated for its similarities to schizophrenia is that of Fragile X syndrome (for example see Kelemen et al., 2013). In Fragile X there is a mutation of multiple CGG repeats on the X chromosome located in an area coding for a protein which appears to be fundamental to healthy brain development and functioning. The phenotypic characteristics of the disorder are developmental delays and cognitive impairments. Additionally, carriers (particularly males) can express psychomotor and cognitive difficulties as they age. Although in children Fragile X is most closely associated with autism spectrum and other pervasive developmental disorders (e.g., Franke et al., 1996; Tsiouris and Brown, 2004), some studies have suggested there may be a link with psychotic disorders. Given the overlapping pathophysiology of dysregulation in BDNF, glutamate and gamma-Aminobutyric acid (GABA) shared by Fragile X and schizophrenia, the potential for common causal pathways seems plausible. A number of studies have also reported associations between elevated rates of schizotypal traits and Fragile X chromosomal abnormalities (Reiss et al., 1988; Kerby and Dawson, 1994; Sobesky et al., 1994; Franke et al., 1996). However, given the levels of social anxiety and withdrawal in those with Fragile X syndrome it would be worthwhile further investigating which aspects of schizotypy are elevated in this syndrome. The overlapping pathophysiology with schizophrenia certainly makes the area of the chromosome affected in Fragile X of significance for those concerned schizophrenia etiology. However, phenotypic presentation, particularly in males, is more developmentally pervasive than the later presentation of symptoms in psychotic disorders. Researchers have linked the expression of Fragile X protein to symptoms and age of onset of schizophrenia (Hossein and Folsom, 2011; Kovács et al., 2013). Therefore, Fragile X protein levels deserve consideration in schizotypal individuals.

Another sex chromosome linked disorder of interest is Klinefelter syndrome where individuals have a 47 XXY karyotype, possessing an extra X chromosome. It occurs in approximately 1 in 670 male births (Bojesen et al., 2003). Many of the genes on the additional X chromosome are inactive which accounts for the relatively mild presentation of difficulties in Klinefelter's compared to other genetic disorders. However, there are some genes on the X chromosome which are homologous with those found on the Y chromosome and consequently are expressed. Males with Klinefelter's tend to be tall in stature, experience difficulties in hormonal expression, experience gynecomastia and infertility. Cognitive deficits in verbal domains (including dyslexia) are most commonly reported (e.g., Fales et al., 2003). The brain structural abnormalities found in Klinefelter's individuals are similar to those reported in patients with schizophrenia, including temporal lobe abnormalities, enlarged ventricles and structurally and functionally reduced or reversed asymmetries (e.g., Reiss et al., 2000; Itti et al., 2003; DeLisi et al., 2005). Approximately, 0.8–1% of male inpatients with schizophrenia are found to have an XXY karyotype (DeLisi et al., 1994). Rates of psychotic symptoms (DeLisi et al., 1994) and psychotic disorders (Bojesen et al., 2006; Cederlöf et al., 2014) are elevated in Klinefelter's individuals compared to the general population (although see Mors et al., 2001). Studies have reported that schizotypal traits are higher in those with Klinefelter's when compared to healthy controls (Van Rijn et al., 2006, 2009; Verhoeven and Egger, 2011). Some authors have suggested that the association between Klinefelter's and schizotypal traits is a function of the cognitive disorganization and verbal deficits found in the genetic syndrome. However, those with Klinefelter's also have elevated diagnoses of autism spectrum, ADHD and bipolar disorders; consequently, the higher schizotypal scores could be an expression of vulnerability to psychopathology in general rather than a demonstrated specificity for psychotic disorders.

Genetic disorders in some ways allow for the possibility of minimizing heterogeneity of genetic abnormalities within a well characterized subpopulation to enable exploration of the consequent phenotypic presentations. However, as yet it is poorly understood how the general genetic background would act to mediate or moderate deletions and insertions. Therefore, the possibility of the effects of epistatic interactions shaping phenotypic expression of genetic disorders needs to be more fully explored. One commonality in presentation of the genetic disorders highlighted above is cognitive deficits. It would be tempting to conclude that the associations with psychotic disorders and schizotypal traits are through a common causal pathway relating to cognitive vulnerabilities in verbal abilities and executive function. However, other developmental disorders such as Downs Syndrome also display cognitive deficits and yet do not have an over representation of psychotic disorders; thus a seemingly simple hypothesis is unsupported given that it cannot be generalized. Further work is needed to determine which aspects of the genetic disorders are particularly predictive of schizotypal traits and whether cognitive deficits, social difficulties or common biological pathways are the crucial factors in determining risk for schizotypal traits.

## Conclusions and future directions

The selection of genetic variations investigated for their association with schizotypal traits has been largely shaped by studies concerned with schizophrenia. These include microsatellite, SNPs, insertions and deletions in neurobiological pathways known to be aberrant in psychotic disorders, including dopamine, glutamate and GABA. The authors were surprised by the relative paucity of papers which considered the genetic underpinnings of schizotypal traits. Unlike the clinical end point of psychotic disorders, schizotypal traits can be readily, cheaply and efficiently characterized using well validated and established measures (Mason, 2015). In contrast to psychotic symptoms, schizotypal traits are stable by virtue of the fact they are defined as a personality construct. Whilst some aspects of the behaviors comprising schizotypal traits may fluctuate, such as the presentation of isolated psychotic symptoms, the core of schizotypal traits will be consistently detectable over multiple time points. Schizotypal traits have been found to be associated with similar environmental factors, such as urbanicity, to those reported for elevating risk for psychotic disorder (e.g., Nelson et al., 2013). Collectively these factors shape a strong argument for considering schizotypal traits as being related to schizophrenia. Many of the studies included in this review have considered schizotypal traits as a whole (although see Mata et al., 2000, 2003). We have referred to schizotypal traits as plural because they comprise a number of behaviors and psychological phenomena. In addition, theorists in this area emphasize either the individual difference or clinical relevance of these traits. These considerations lead to differences in the measures used and whether differing dimensions (positive, negative, unusual experiences, cognitive disorganization, interpersonal) are emphasized. It is possible that these differences in measurement account for some of the inconsistencies in genetic association studies. Indeed, some of the dimensions of schizotypal traits could be more likely to be underpinned by stable genetic factors. For instance, disorganization and interpersonal schizotypal traits have a stability to them which suggests biological underpinnings are more plausible. Whilst a propensity toward unusual perceptual experiences may be further “down stream” of genetic influences, given that they are likely triggered by factors such as stress or substance use. Linkage studies have not as yet provided evidence for the genomic components which confer risk for schizotypal traits within families. A gene-dosing relationship is implied between psychotic disorders and schizotypal traits, given that there are elevated levels in relatives of patients with schizophrenia compared to the general population. This suggests further investigation of the biological correlates of schizotypal traits is warranted.

However, whether these investigations should be exclusively guided by the pathophysiology of schizophrenia could be a matter of debate. Given the assumption that schizotypal traits exist on a continuum with psychotic disorders, it seems likely that at least some of the genetic liability for schizophrenia should underpin schizotypal traits; yet, this is fraught with difficulties. Schizotypal individuals will likely possess some of the genetic liability for psychotic disorders but also some biological protective factors which assist in preventing transition to a diagnosable psychotic disorder. Although we recognize that risk for psychotic disorders is an interaction between genetic and environmental factors, existing evidence points to a strong role for genetic and biological vulnerability. In addition, it is possible that schizotypal traits present a broad phenotype for the liability to serious mental health disorders in general rather than psychotic disorders *per se*. The evidence accumulated here from genetic disorders may provide intriguing evidence for this. Schizotypal traits may be elevated in genetic disorders which over express psychotic disorders, however, these same genetic disorders also over represent pervasive developmental disorders, ADHD and bipolar disorder.

The authors could not find any papers which examined the interaction between genes and the environment for schizotypal traits. From a theoretical perspective this may be due to the assumed stability in a personality trait. A longitudinal study could track the effects of childhood events on the differing trajectories theorized to occur with schizotypal traits in response to environmental factors (Raine, 2006). Alternatively, shorter longitudinal follow up periods could reveal environmental factors in adulthood which exacerbate the presentation of schizotypal traits. These studies could consider the effects of particular genetic variations highlighted in this review. Such a design also has the potential to identify protective as well as risk genes. The investigation of gene-environment interactions on schizotypal traits is certainly an area for development. There are a number of papers considering gene-environment interactions for psychotic symptoms (e.g., Alemany et al., 2011; Ramsay et al., 2013). This perhaps suggests a way forward may be to refine the measurement of the schizotypal phenotype with the addition of state-like measures capturing psychotic symptoms separately. In this way gene-environment interactions could investigate effects along the psychosis continuum for expression of psychotic symptoms against a background risk of schizotypal traits. Whilst theoretically robust this is statistically complex and would require a relatively large sample size in order to have sufficient power to detect what, will likely be small effects.

The genetic disorders reviewed highlight the potential role for cognitive deficits being an additional factor for psychosis risk. From the perspective of investigation of genetic underpinnings, cognitive performance has many appealing features. It is objectively definable on the basis of performance rather than self-report; and, it is closer to the expression of genes than collections of (personality) behaviors, given that it can be linked to particular brain areas. Cognitive performance has been linked more consistently to genetic variation (e.g., Park and Waldman, 2014; Heck et al., 2014). Cognitive deficits can be linked to the structural brain abnormalities reported in genetic disorders. This provides a link from well characterized genetic polymorphisms to changes in brain structure which could underpin the cognitive deficits found in genetic disorders. Brain structure abnormalities have been found in those with schizotypal traits who are otherwise healthy individuals (e.g., Smallman et al., 2014). Therefore, in those characterized for both their schizotypal and genetic profiles, we could examination associations between carefully selected genetic polymorphisms, gray and white matter density and cognitive performance. In this way, research can begin to close the gap between the neurodevelopmental effects of the gene, their cognitive outcomes and whether these differ with schizotypal traits. Cognitive deficits are found across a number of genetic and mental health disorders; consequently, they do not represent an alternative single end point for genetic studies. Rather they offer a way to refine a complex and heterogeneous phenotype like schizotypy. For instance, schizotypes who have poorer cognitive performance could represent a subtype that may be genetically enriched for risk. Distinguishing their genetic profile from schizotypes with intact cognitive performance could, therefore, begin to tease out the complexities of psychosis liability where we see multiple factors converging.

The genetic underpinnings of schizotypal traits have received limited attention; there has been substantial focus on general psychosis risk rather than an emphasis on schizotypal traits specifically. As such there is the opportunity for further investigation and exploration. Using the investigations of psychotic disorders represents a start point for identification of genes of interest for schizotypal traits but focus needs to be widened. Investigations now are examining the genes associated with particular symptoms with minimal attention to diagnostic category (e.g., Craddock et al., 2009; Hamshere et al., 2009). This broader scope represents a way forward for the investigation of schizotypal traits. As such, perhaps we need to consider the genetic underpinnings of the differing dimensions of schizotypal traits rather than focusing on the intermediate phenotype as a whole. Given that schizotypy is heterogeneous, more refinement of the clinical end point of interest needs considering; the inclusion of a cognitive or psychotic symptom measure could help to narrow the specificity for genetic association studies.

## Author contributions

EW Contribution to the conception and design of the review, literature search for acquisition of studies, drafting of section 2.1 and 3 of the manuscript, critical review, editing, and formatting of the full manuscript and final approval of the version to be submitted. FF Contribution to the conception and design of the review, literature search for section 2.2, drafting of section 2.2, and 3, Critical review of manuscript and final approval of the version to be submitted. MS Contribution to the conception and design of the review, literature search for acquisition of studies for inclusion in the review, drafting of section 3, and final approval of the version to be submitted. EB Contribution to the conception and design of the review, literature search for acquisition of studies, drafting of sections 1, 4, and 5. Critical review of sections 2.1, 2.2, and 3. Editorial review of full manuscript and final approval of the version to be submitted. All authors agree to be accountable for all aspects of the work

## Funding

EB is funded by the NARSAD Young Investigator Award, in kind support from GW Pharmaceuticals, and two internal grants for the University of Wollongong FF is funded by an NHMRC grant.

### Conflict of interest statement

The authors declare that the research was conducted in the absence of any commercial or financial relationships that could be construed as a potential conflict of interest.
